# POLYGENIC RISK SCORES FOR ALZHEIMER’S DISEASE AND GENERAL COGNITIVE FUNCTION ARE ASSOCIATED WITH MEASURES OF COGNITION IN OLDER SOUTH ASIANS

**DOI:** 10.1093/geroni/igae098.0564

**Published:** 2024-12-31

**Authors:** Gustavo Duque

**Affiliations:** Bone, Muscle & Geroscience Group, Research Institute of the McGill University Health Centre, Montreal, Quebec, Canada

## Abstract

TBD

